# Association between breastfeeding duration, non-nutritive sucking habits and dental arch dimensions in deciduous dentition: a cross-sectional study

**DOI:** 10.1186/s40510-014-0059-4

**Published:** 2014-10-31

**Authors:** Shiv Shankar Agarwal, Karan Nehra, Mohit Sharma, Balakrishna Jayan, Anish Poonia, Hiteshwar Bhattal

**Affiliations:** Division of Orthodontics and Dentofacial Orthopedics, Department of Dental Surgery, Armed Forces Medical College, Pune, 411040 India; Army Dental Centre (R&R), New Delhi, India; CMDC (CC), Pune, India

**Keywords:** Breastfeeding duration, Posterior crossbite, Anterior open bite, Non-nutritive sucking, Dental arch diameters

## Abstract

**Background:**

This cross-sectional retrospective study was conducted to determine association between breastfeeding duration, non-nutritive sucking habits, dental arch transverse diameters, posterior crossbite and anterior open bite in deciduous dentition.

**Methods:**

415 children (228 males and 187 females), 4 to 6 years old, from a mixed Indian population were clinically examined. Based on written questionnaire answered by parents, children were divided into two groups: group 1 (breastfed for <6 months (n = 158)) and group 2 (breastfed for ≥6 months (n = 257)). The associations were analysed using chi-square test (P < 0.05 taken as statistically significant). Odds ratio (OR) was calculated to determine the strength of associations tested. Multivariate logistic regression analysis was done for obtaining independent predictors of posterior crossbite and maxillary and mandibular IMD (Inter-molar distance) and ICD (Inter-canine distance).

**Results:**

Non-nutritive sucking (NNS) was present in 15.18% children (20.3% in group 1 as compared to 12.1% in group 2 (P = 0.024)). The average ICD and IMD in maxilla and average IMD in mandible were significantly higher among group 2 as compared to group 1 (P < 0.01). In mandible, average ICD did not differ significantly between the two groups (P = 0.342). The distribution of anterior open bite did not differ significantly between the two groups (P = 0.865). The distribution of posterior crossbite was significantly different between the two groups (P = 0.001). OR assessment (OR = 1.852) revealed that group 1 had almost twofold higher prevalence of NNS habits than group 2. Multivariate logistic regression analysis revealed that the first group had independently fourfold increased risk of developing crossbite compared to the second group (OR = 4.3). Multivariate linear regression analysis also revealed that age and breastfeeding duration were the most significant determinants of ICD and IMD.

**Conclusions:**

An increased prevalence of NNS in the first group suggests that NNS is a dominant variable in the association between breastfeeding duration and reduced intra-arch transverse diameters which leads to increased prevalence of posterior crossbites as seen in our study. Mandibular inter-canine width is however unaffected due to a lowered tongue posture seen in these children.

## Background

The oral and peri-oral musculature especially the lips, cheeks and tongue are well developed in a newborn. Apart from affecting the harmonious growth and development of the jaws, they also guide occlusal development and help in establishing a correct inter-maxillary relationship. Suckling done by the child during breastfeeding is more physiologic and promotes a better oro-facial development. In the literature, an increased duration of breastfeeding has often been associated with a reduced incidence of malocclusion [1,2].

Apart from oro-facial implications, reduced breastfeeding duration in a child has also been associated with inferior health, delayed psychological development and inferior immunological status as compared to the adequately breastfed counterparts [3]. Breastfeeding stimulates normal craniofacial growth and development and prevents the child from indulging in non-nutritive sucking habits [4]. The World Health Organization recommends minimum exclusive maternal breastfeeding up to the age of 6 months [5].

The deciduous dental arches lay the foundation on which proper development of the permanent dental arches takes place. The inter-canine and inter-molar widths are largely established during the deciduous dentition and do not increase drastically during growth and development. Various authors have analysed the association of inter-canine and inter-molar width with the presence of various deleterious oral habits in children [6]. Non-nutritive sucking like pacifier, digit or dummy sucking has often been implicated as an important etiologic factor in the development of posterior crossbites [7]. Heredity, nasal obstruction (caused by enlarged tonsils and adenoids) and oral breathing have also been associated with the increased prevalence of posterior crossbites [8].

This altered occlusal development may be due to genetic or epigenetic factors, and the literature is still controversial in this regard. A few authors found no significant relationship between breastfeeding and the development of malocclusion [9-11].

### Objectives

Keeping the above-mentioned factors in mind and also that the deciduous dentition is an optimum phase to promote various preventive and interceptive measures, a study was designed to analyse the association between (1) exclusive breastfeeding duration and the prevalence non-nutritive sucking (NNS) habits, (2) breastfeeding duration and the prevalence of posterior crossbites, (3) breastfeeding duration and the prevalence of anterior open bites, (4) breastfeeding duration and intra-arch transverse diameters (inter-canine distance (ICD) and inter-molar distance (IMD)) in the deciduous dentition and (5) NNS and all the above-mentioned parameters.

## Methods

### Study design

This is a cross-sectional retrospective study conducted to achieve the objectives as mentioned above.

### Setting

The study was conducted in the Division of Orthodontics and Dentofacial Orthopedics, Armed Forces Medical College, Pune, India. Ethical approval was obtained from the institutional ethical committee. Informed consent forms were distributed to the parents of the children, and those unwilling to participate were excluded from the study.

### Participants

Six hundred and fifty written questionnaires were distributed to the parents of children studying at two randomly selected primary schools in Pune. The questionnaires were framed based upon inclusion and exclusion criteria for this study and also included information on age, sex, height and weight of the children. The medical history of the mother during pregnancy, the type of delivery and the length of gestation were recorded. Information on NNS was also requested in the questionnaires. Only full-term children delivered normally were included in the study to further ensure a healthy sample selection. The inclusion criteria were children aged 4 to 6 years and studying at one of the schools selected for this study; presence of a normal number, size and shape of teeth; absence of extensive caries or large restorations and teeth with hopeless prognosis; parental completion of the questionnaire and absence of any local or systemic condition that might affect the development of bones and teeth. The exclusion criteria were children not studying in the schools selected for this study; children not matching our age group; parental refusal to fill in the written questionnaire; children with extensive caries and large restorations; presence of hopeless teeth; children with missing, supernumerary or malformed teeth and presence of any local or systemic condition affecting the development of bones and teeth.

### Variables

The clinical examination was performed by two orthodontists. The parameters measured were anterior open bite, posterior crossbite and maxillary and mandibular IMD and ICD.

### Measurements

Alginate impressions of the maxillary and mandibular dental arches were taken, study models were prepared and the occlusal relationships were recorded. Posterior crossbite was diagnosed when a reverse occlusal relationship was observed in the transverse plane between at least one posterior tooth (i.e. deciduous canine or molar) [4,12]. Anterior open bite was defined as the absence of vertical overlap between the upper and lower teeth in the anterior region.

Fine-pointed callipers which were accurate to within 0.01 mm were used for the measurement of the arch widths on the study models. The inter-canine distance was taken as the distance between the deciduous canine cusp tips or the estimated location of the cusp tip if wear facets were present. The inter-molar distance was taken as the distance between the mesiobuccal cusp tips of the deciduous second molars.

### Bias

The schools and the subjects were randomly selected to avoid selection bias. Both the examiners were blinded for the breastfeeding status of participants at the time of clinical examination to avoid observer bias.

### Study size

Based on the inclusion and exclusion criteria, 415 children (228 males and 187 females) were selected for this study. The remaining 235 children who did not meet the inclusion criteria were excluded from the study. The main reason for this huge exclusion was children not matching our age group (150 children); 45 children had one of their deciduous molar or canine missing, 25 children had extensive caries with mutilation of the tooth structure in their deciduous molar or canine and parents of 15 children refused to participate in the study because of social commitments.

### Quantitative variables

Based on the questionnaires answered by the parents, a retrospective investigation was made concerning the duration for which the children were exclusively breastfed. The children were divided into two groups: group 1 (breastfed for <6 months (*n* = 158)) and group 2 (breastfed for ≥6 months (*n* = 257)) (Table 1). The data regarding the duration of breastfeeding and NNS was collected and recorded in Microsoft Excel Worksheet 2010. As per the WHO guideline [5], the minimum recommended breastfeeding duration of 6 months or more was taken into consideration.

**Table 1:** **The distribution of various parameters studied according to the duration of breastfeeding**

**Parameters**	**Category**	**Breastfeeding <6 months (** ***n*** **= 158)**	**Breastfeeding ≥6 months (** ***n*** **= 257)**	**OR (95% CI)**	***P*** **value**
Age (years)	4	0	1 (0.4)	--	--
	5	61 (38.6)	80 (31.1)	--	
	6	97 (61.4)	176 (68.5)	--	
Sex	Male	85 (53.8)	143 (55.6)	--	--
	Female	73 (46.2)	114 (44.4)	--	
Sucking habit	Digit sucking	17 (10.8)	14 (5.4)	2.093 (1.00 to 4.37)	0.046 (S)
	Thumb sucking	12 (7.6)	13 (5.1)	1.543 (0.68 to 3.47)	0.292 (NS)
	Dummy sucking	4 (2.5)	4 (1.6)	1.643 (0.40 to 6.66)	0.483 (NS)
	Non-nutritive sucking	32 (20.3)	31 (12.1)	1.852 (1.08 to 3.18)	0.024 (S)
Malocclusion	Open bite	1 (0.6)	2 (0.8)	0.812 (0.073 to 9.03)	0.865 (NS)
	Crossbite	20 (12.7)	5 (1.9)	7.304 (2.68 to 19.89)	0.001 (S)
Distance (cm)	Maxilla ICD	3.96 ± 0.33	4.05 ± 0.34	--	0.006 (S)
	Maxilla IMD	5.08 ± 0.34	5.19 ± 0.31	--	0.001 (S)
	Mandible ICD	3.53 ± 0.33	3.56 ± 0.32	--	0.342 (NS)
	Mandible IMD	4.63 ± 0.30	4.74 ± 0.34	--	0.002 (S)

Values of age, sex, sucking habit and malocclusion are *n* (%), *P* values of which are obtained using a chi-square test. Values of distance are shown as mean ± standard deviation, *P* values of which are obtained using independent samples ‘*t*’ test after confirming the underlying normality assumption. *P* < 0.05 is considered to be statistically significant. S, statistically significant; NS, statistically non-significant.

### Statistical methods

Statistical analyses were performed using the software Statistical Package for the Social Sciences (SPSS 11.5; SPSS Inc., Chicago, IL, USA) for MS Windows. A Pearson chi-square test was performed to verify the associations. *P* values less than 0.05 were considered as statistically significant. In order to measure the strength of the associations tested, the odds ratio (OR) (with 95% confidence interval (CI)) was calculated. Multivariate logistic regression analysis was performed for obtaining the independent determinants of posterior crossbite and maxillary and mandibular IMD and ICD. The Cohen kappa test was done for the assessment of inter-examiner reliability.

## Results

There was a considerable agreement in the assessment of clinical parameters (open bite, crossbite, maxillary and mandibular ICD and IMD) between the two orthodontists (kappa value = 0.758, *P* = 0.001).

The age and sex distributions showed that majority of children were in the age groups of 5 and 6 years (34% and 65.8%, respectively). Among them, 54.9% were males and 45.1% were females; 38.07% of children were breastfed <6 months and 61.92% were breastfed ≥6 months (Table 1).

**Figure 1:**
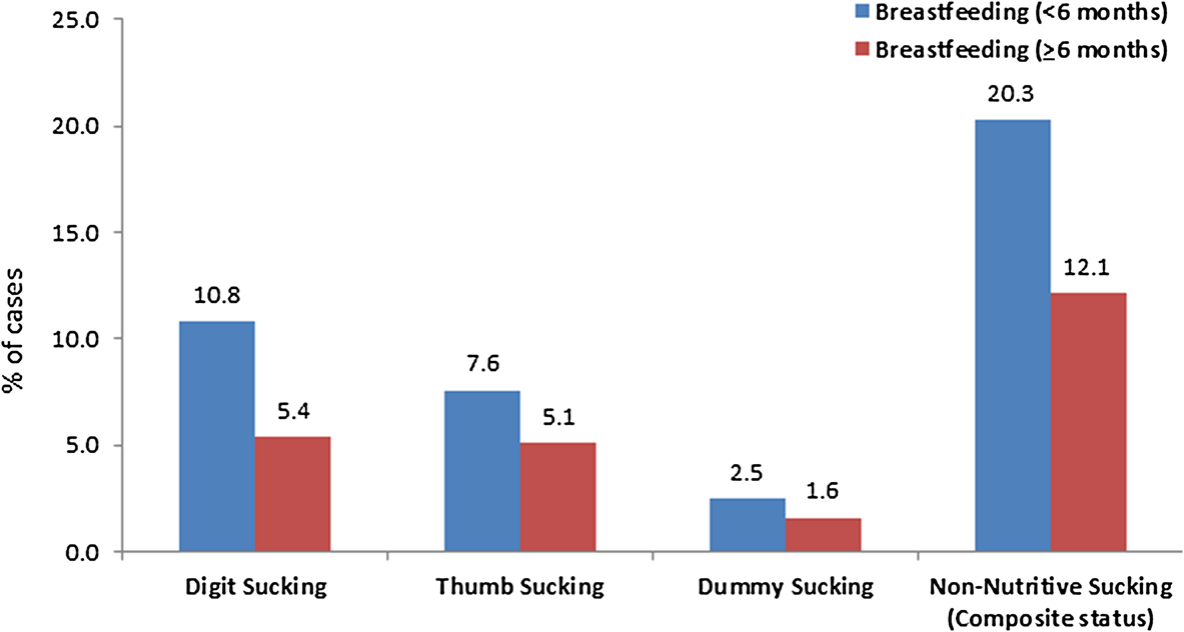
**The distribution of non-nutritive sucking habits according to the duration of breastfeeding.**

NNS habits were present in 15.18% children. In children breastfed <6 months, 20.3% were indulged in NNS habits while the frequency was 12.1% in children breastfed ≥6 months. The distribution of digit sucking differed significantly between the two groups. The children who were breastfed <6 months had a higher prevalence of digit sucking habit compared to the control group. The difference was statistically significant (*P* = 0.046). The distribution of thumb sucking and dummy sucking did not differ significantly between the two breastfeeding groups (*P* > 0.05). The distribution of non-nutritive sucking (i.e. the composite status of digit sucking, dummy or pacifier sucking) was significantly different between the two groups (*P* = 0.024). The children who were breastfed <6 months had a higher prevalence of NNS compared to the control group. The difference was statistically significant (*P* < 0.05) (Tables 1 and 2, Figure 1).

**Table 2:** **The distribution of malocclusion according to non-nutritive sucking habits**

**Parameters**	**Sucking habit (** ***n*** **= 63)**	**No sucking habit (** ***n*** **= 352)**	***P*** **value**
Breastfeeding <6 months			
Open bite	1 (3.1)	0	0.203 (NS)
Crossbite	0	20 (15.9)	0.014 (S)
Breastfeeding ≥6 months			
Open bite	0	2 (0.9)	0.999 (NS)
Crossbite	1 (3.2)	4 (1.8)	0.477 (NS)
Overall			
Open bite	1 (0.5)	2 (2.5)	0.379 (NS)
Crossbite	1 (3.8)	24 (21.2)	0.108 (NS)

Values are *n* (% of cases). *P* value was obtained using a chi-square test. *P* < 0.05 was considered to be statistically significant. Non-nutritive sucking is a composite status of digit, thumb and dummy sucking. S, statistically significant; NS, statistically non-significant.

**Figure 2:**
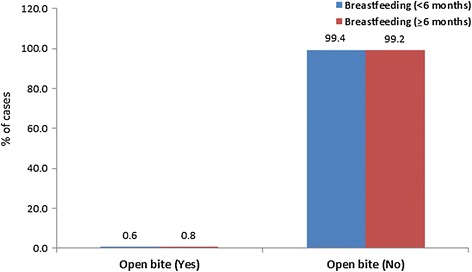
**The distribution of open bite according to the duration of breastfeeding.**

The distribution of anterior open bite did not differ significantly between the two groups (*P* = 0.865). Only one child in the first group (0.6%) and two in the second group (0.8%) had an anterior open bite (Table 1, Figure 2).

**Figure 3:**
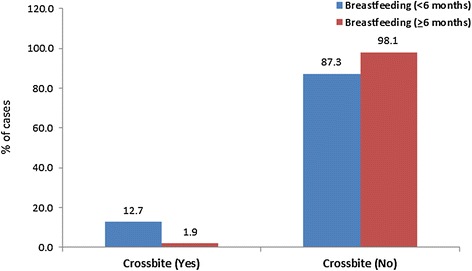
**The distribution of crossbite according to the duration of breastfeeding.**

The distribution of crossbite showed a significant difference between the two breastfeeding groups (*P* = 0.001); 12.7% in the first group and only 1.9% in the second group had posterior crossbites (Table 1, Figure 3).

No statistically significant association was observed between NNS habits and the prevalence of posterior crossbites (*P* = 0.108) and anterior open bites (*P* = 0.379). The OR revealed that children breastfed for <6 months had almost twofold prevalence of NNS habits than children who were breastfed ≥6 months (Tables 1 and 2).

**Figure 4:**
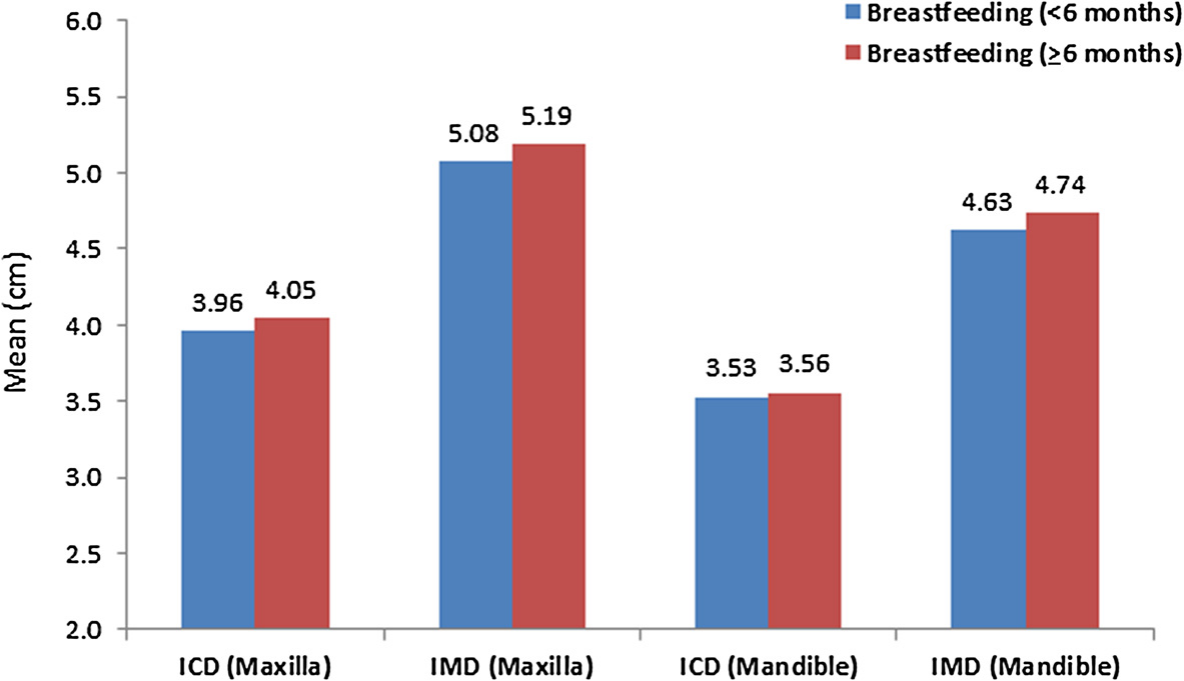
**The distribution of inter-canine (ICD) and inter-molar (IMD) distances according to the duration of breastfeeding.**

The average ICD in the maxilla was significantly higher among the second group as compared to the first group (*P* = 0.006). However, in the mandible, the average ICD did not differ significantly between the two breastfeeding groups (*P* = 0.342). The average IMD in both the maxilla and mandible was significantly higher among the second group as compared to the first group (*P* < 0.01) (Table 1, Figure 4).

The males showed greater mean ICD and IMD in the maxilla than the females, and this association was statistically significant (*P* < 0.01). No such significant association was seen in the mandible (Table 3). A statistically significant association was seen in the distribution of inter-canine and inter-molar distance and the age of the children (Table 4).

**Table 3:** **The distribution of inter-canine and inter-molar distances according to sex**

	**Maxilla**		**Mandible**	
	**ICD (cm)**	**IMD (cm)**	**ICD (cm)**	**IMD (cm)**
Male (*n* = 228)	4.05 ± 0.35	5.19 ± 0.33	3.56 ± 0.32	4.73 ± 0.32
Female (*n* = 187)	3.97 ± 0.32	5.10 ± 0.32	3.54 ± 0.33	4.66 ± 0.34
*P* value	0.024 (S)	0.007 (S)	0.446 (NS)	0.065 (NS)

Values are mean ± standard deviation. *P* value was calculated by independent samples ‘*t*’ test after confirming the underlying normality assumption. *P* < 0.05 was considered to be statistically significant. ICD, inter-canine distance; IMD, inter-molar distance; S, statistically significant; NS, statistically non-significant.

**Table 4:** **The distribution of inter-canine and inter-molar distances according to age group**

	**Maxilla**		**Mandible**	
	**ICD (cm)**	**IMD (cm)**	**ICD (cm)**	**IMD (cm)**
4 years (*n* = 1)	4.10 ± 0.0	5.10 ± 0.0	3.60 ± 0.0	4.50 ± 0.0
5 years (*n* = 141)	3.93 ± 0.29	5.06 ± 0.33	3.47 ± 0.27	4.64 ± 0.31
6 years (*n* = 273)	4.06 ± 0.35	5.20 ± 0.32	3.59 ± 0.35	4.73 ± 0.33
*P* value	0.001 (S)	0.001 (S)	0.002 (S)	0.018 (S)

Values are mean ± standard deviation. *P* value was obtained by one-way analysis of variance (ANOVA) after confirming the underlying normality assumption. *P* < 0.05 was considered to be statistically significant. ICD, inter-canine distance; IMD, inter-molar distance; S, statistically significant; NS, statistically non-significant.

The multivariate logistic regression analysis done for obtaining the independent determinants of crossbite revealed that the prevalence of posterior crossbite was independently and significantly determined by lesser breastfeeding duration only (*P* < 0.001). The cases that had breastfeeding <6 months had independently fourfold increased risk of developing posterior crossbite as compared to the cases that had ≥6 months breastfeeding. Age, sex and NNS habit did not independently determine the occurrence of posterior crossbite (Table 5).

**Table 5:** **Multivariate logistic regression analysis for obtaining the independent determinants of crossbite**

**Variables included in the model**		**Odds ratio (OR)**	**95% CI for odds ratio**	***P*** **value**
Age	<6 years	1.0	--	--
	≥6 years	1.3	0.4 to 1.8	0.213 (non-significant)
Sex	Male	1.0	--	--
	Female	1.2	0.3 to 1.5	0.116 (non-significant)
Breastfeeding duration	≥6 months	1.0	--	--
	<6 months	4.3	2.4 to 6.5	0.001 (significant)
Non-nutritive sucking habit	No	1.0	--	--
	Yes	1.4	0.6 to 1.9	0.108 (non-significant)

[odds ratio = 1: reference category].

The multivariate logistic regression analysis done for obtaining the independent predictors of maxillary and mandibular ICD and IMD revealed that age, sex and breastfeeding duration were the independent and significant predictors of IMD (maxilla) (adjusted *R*^2^ = 7.3%, *P* < 0.001). Age and breastfeeding duration were the independent and significant predictors of IMD (mandible) (adjusted *R*^2^ = 3.7%, *P* < 0.001). Age, sex and breastfeeding duration were the independent and significant predictors of ICD (maxilla) (adjusted *R*^2^ = 4.7%, *P* < 0.001). Age alone is the independent and significant predictor of ICD (mandible) (adjusted *R*^2^ = 2.2%, *P* < 0.01) (Table 6).

**Table 6:** **Multivariate linear regression for finding the independent predictors of IMD and ICD measurements**

**Variables in the model**	**Outcome measure**	**Standard beta**	**Adjusted** ***R*** ^**2**^ **(%)**	***P*** **value**
Constant	IMD (maxilla)	--	7.3	0.001
Age		0.180		0.001
Sex		−0.114		0.017
Breastfeeding duration		0.168		0.001
Non-nutritive sucking habit		0.003		0.949
Constant	IMD (mandible)	--	3.7	0.001
Age		0.122		0.012
Sex		−0.078		0.110
Breastfeeding duration		0.143		0.003
Non-nutritive sucking habit		0.008		0.873
Constant	ICD (maxilla)	--	4.7	0.001
Age		0.161		0.001
Sex		−0.096		0.047
Breastfeeding duration		0.119		0.014
Non-nutritive sucking habit		0.025		0.609
Constant	ICD (mandible)	--	2.2	0.001
Age		0.167		0.001
Sex		−0.023		0.638
Breastfeeding duration		0.034		0.495
Non-nutritive sucking habit		0.014		0.781

All outcome measures are normally distributed.

## Discussion

According to the functional matrix hypothesis, form and function are inter-related, i.e. the growth and development of oro-facial structures is dictated by various functions performed by the stomatognathic system [13]. A newborn has a well-developed suckling reflex which is important for suckling milk from the mother's breasts. A reduced duration of breastfeeding leads to the child's indulgence in various NNS habits, and disturbance of oro-facial equilibrium takes place which has been associated with various malocclusions [14].

The premaxillary region of the child is stimulated by the upward and outward movements of the tongue on the mother's breasts during suckling while the mandibular movements during suckling are thought to promote optimal jaw growth, whereas during non-nutritive sucking, the posteriorly acting forces of the buccinator muscle act against the forward acting forces of suckling during breastfeeding and may restrain jaw growth [15]. These NNS habits soothe the infants, reduce their teething discomforts and are believed to relax them during stressful situations [14].

Whether genetics is the main decisive factor in the development of malocclusion or various epigenetic stimuli cause these phenotypic alterations in the genetic pattern of growth and development of oro-facial structures through neuromuscular adaptation has always been controversial in the literature [13,16-19].

In our study, the prevalence of NNS habits was significantly higher in children who were breastfed ≤6 months. The deleterious effects of NNS habits on the dental arches have been reported previously in the literature, and our results are in concurrence with these studies [4,10,12,20,21].

The mean maxillary and mandibular inter-canine diameters reported in our study were similar to those observed by Aznar et al. [6] in their study on Spanish children in which they observed no statistically significant association between dental arch widths and the prevalence and duration of breastfeeding. They observed a significant reduction in maxillary IMD in children with increased bottle feeding duration, and a non-uniform increase in mandibular ICD was seen with the increase in duration of the habit. In contrast to this, in our study, we observed a significant reduction in maxillary and mandibular IMD and maxillary ICD in children who were breastfed <6 months. However, no statistically significant association could be obtained between mandibular ICD and the duration of breastfeeding in our study. Greater ICD and IMD were observed in males compared to females in both the arches in our study which was in agreement with their study.

Our findings are also similar to those of Larsson [4,22] and Ogaard et al. [10] who observed that increased duration of dummy sucking was associated with a reduced maxillary ICD and an increased mandibular ICD which they considered to be due to low tongue position during sucking. However, in our study, no statistically significant difference in mandibular ICD was observed between the two breastfeeding groups.

Malandris and Mahoney [23] and Larsson [4] in their studies related the increased incidence of posterior crossbite with early weaning, and they considered it to be due to the altered oro-facial muscle activity associated with bottle feeding. These findings were similar to those of our study. In our study, the prevalence of crossbites was significantly higher in children who were breastfed <6 months. Also, a statistically significant association was observed between NNS and posterior crossbites. Our study findings were opposite to those of Warren and Bishara [11] who did not find any significant difference in transverse dental arch parameters between children with or without NNS habits and between those who were breastfed longer and those breastfed for shorter duration or no breastfeeding.

Johnson and Larsson [24] in their study, apart from observing several social and cultural factors presumed to be risk factors for the development of NNS habits in a child, also observed an association between increased breastfeeding duration and protection against sudden infant death syndrome and higher occurrence of otitis media in children indulged in NNS habits.

It can also be hypothesised from our study findings that NNS habits act as a dominant variable in the relationship between breastfeeding duration and reduced intra-arch transverse dimensions and occurrence of posterior crossbites and anterior open bites. According to our study interpretation, if the breastfeeding duration is <6 months, there is a twofold likelihood that the child would develop a non-nutritive sucking habit which may further alter his/her intra-arch transverse dimensions usually leading to posterior crossbites, and the sucking habits may also lead to anterior open bites. Multivariate logistic regression analysis was done in our study to obtain the independent determinants of crossbite which revealed that the less breastfed group had independently fourfold increased risk of developing crossbite compared to the control group (OR = 4.3). Multivariate logistic regression analysis was also done for obtaining the independent predictors of maxillary and mandibular ICD and IMD. However, the *R*^2^ values (%) which explain the amount of variance of the outcome variable are relatively low in our study for all outcome measures, and this is a clear limitation of our multivariate findings. This means that there are other factors also which strongly correlate with the ICD and IMD measurements in addition to the ones we have measured in the present study. Therefore, further prospective studies with a larger sample size are required to enhance our understanding in this regard.

### Limitations

This cross-sectional retrospective study has the following limitations:

1. The cross-sectional design of this study is associated with bias such as recall bias, i.e. accuracy of reporting of breastfeeding status.
2. The exact duration of breastfeeding and frequency of NNS habits were not reported, which is another limitation of this study.
3. The *R*^2^ values (%) are relatively low in our study for all outcome measures, and this is a clear limitation of our multivariate findings.

## Conclusions

The following conclusions could be drawn from our study:

1. An association was observed between reduced breastfeeding duration (<6 months) and higher prevalence of NNS habits.
2. An association was also observed between reduced breastfeeding duration (<6 months) and increased prevalence of posterior crossbites and reduced intra-arch transverse dimensions particularly in the maxilla.
3. An association was also observed between the presence of NNS habits and increased prevalence of posterior crossbites and reduced intra-arch transverse dimensions particularly in the maxilla.
4. NNS habits may act as a dominant variable between reduced breastfeeding duration and reduced intra-arch transverse dimensions and increased prevalence of posterior crossbites.
5. A child breastfed for <6 months had a twofold likelihood that he/she would develop a non-nutritive sucking habit.
